# Study on Structure–Function Integrated Polymer-Based Microwave-Absorption Composites

**DOI:** 10.3390/polym16172472

**Published:** 2024-08-30

**Authors:** Jiaqu Zhang, Zexu Fan, Bo Li, Dengxun Ren, Mingzhen Xu

**Affiliations:** School of Materials and Energy, University of Electronic Science and Technology of China, Chengdu 610054, China; 19908094739@163.com (J.Z.); fanzexu150@163.com (Z.F.); uestc_lb87@163.com (B.L.)

**Keywords:** resin matrix, fiber reinforced, wave-absorbing properties, absorbing mechanism, structural composites

## Abstract

This article provides an in-depth exploration of the current state of research in microwave-absorbing composite materials, juxtaposing the status quo of coating type and structurally reinforced resin-based composites, with a particular emphasis on the latter’s structural and performance superiority. It succinctly elucidates the mechanisms of electromagnetic shielding, as well as the conditions and underlying principles that govern the absorption of microwaves by composite materials. The review continues by dissecting the strategies for enhancing the microwave-absorption capabilities of resin-based composites, including the classification of absorbents, absorbent selection, and an overview of structural design innovations in microwave-absorbing materials. Structural wave-absorbing composites are manufactured by combining different types of resin matrices, absorbers, reinforcing fibers and construction methods. The interactions between these components are scrutinized to reveal how each contributes to the overall performance of the composite. We spotlight the unique construction methods and the intricate relationship between structure and performance in structurally reinforced composites, offering insights into the optimization strategies for composite-material absorption characteristics. Concluding with a forward-looking perspective, the article contemplates the burgeoning potential and future applications of fiber-reinforced resin-based microwave-absorbing composites, setting the stage for a new era in material science and technology.

## 1. Introduction

With the swift progression of contemporary technology, electronic devices and state-of-the-art wireless communication technologies have infiltrated the commercial, civilian, and military sectors with increasing ubiquity. These innovations, while offering unparalleled convenience, have also engendered a plethora of challenges, especially within the military arena. To bolster the concealment capabilities of military armaments and aerial assets, the advent of stealth technology has become paramount. Stealth technology pertains to the capacity to elude detection by radar and other surveillance mechanisms within a confined range, with its potency fundamentally predicated on the electromagnetic wave-absorption characteristics of the materials employed [1]. Reflectance loss is a pivotal metric for assessing the efficacy of microwave-absorbing materials, where a diminished value, coupled with an expansive bandwidth, demarcates exceptional absorption characteristics. A threshold below −10 dB in reflectance loss ensures the absorption of over 90% of incident electromagnetic waves, thereby underscoring the material’s proficiency in microwave absorption. Moreover, an extensive bandwidth that maintains reflectance loss beneath this threshold extends the electromagnetic wave-absorbing material’s absorption band, thereby enhancing its applicability and effectiveness in broader frequency ranges. Materials with absorption properties are bifurcated into two categories based on their fabrication methodologies: the coating type and the structural type. The coating-type absorbers entail the application of an absorptive coating onto the surface of the object and are heralded for their straightforward production process, cost-effectiveness, and malleability in adjustment. Nonetheless, they are marred by limitations, such as a restricted absorption bandwidth, a propensity for detachment, and a substantial increment in weight. Conversely, structural-type absorbers exhibit enhanced absorptive and load-bearing attributes without incurring additional weight to the material. Composite materials that integrate structural and stealth functionalities are a genre of structural absorbers and have emerged as a pivotal area of inquiry for forthcoming research. 

Structural-stealth integrated composites, specifically fiber-reinforced resin-based wave-absorbing composites, epitomize a harmonious amalgamation of load-bearing attributes and adjustable wave-absorptive capabilities. This is achieved through the modulation of structural configurations and absorbent materials, culminating in an integrated functional quality of structure and wave absorption. In the quest to advance these composites, the choice of reinforcing fibers, the formulation of the resin matrix and absorbent materials, and the architectural design are pivotal factors that significantly influence their performance. Resin-based microwave absorbers are esteemed for their cost-effectiveness, reduced density, facile fabrication, and adjustable mechanical and electromagnetic wave-absorption characteristics. Polyimide (PI) resins, with their innate superiority in thermal and oxidative resistance, coupled with robust dielectric and mechanical properties, serve as exemplary matrix materials [2]. Kevlar fibers, frequently employed as the reinforcing phase, not only bolster the composite’s mechanical integrity but also endow it with advantageous wave-transmissive qualities. Sophisticated multi-layered or multi-core structural configurations, such as honeycomb, foam, and corrugated-core sandwich designs, are instrumental in enhancing both the absorptive and mechanical characteristics of the material ensemble. The B-2 bomber’s airframe and wing skin are illustrative of this paradigm, utilizing a honeycomb core structure for wave absorption, Kevlar fiber-reinforced epoxy resin as the composite overlay, a honeycomb core at the center, and a graphite-reinforced epoxy-resin composite as the underlying substrate. This exemplar underscores that the judicious selection of the resin matrix and absorbent materials, complemented by an astute structural design, are the crux of the research into structural-stealth integrated composite materials [3]. This study investigates the mechanical and microwave-absorption properties of composite materials, fabricated through the intricate assembly of diverse absorbents, fibers, and resin matrices. By dissecting the construction techniques and the synergistic relationship between structure and functionality, we unveil the nuances of electromagnetic wave absorption. This lays the groundwork for the development of hybrid composite structures, opening avenues for the innovation of advanced microwave-absorbing materials with tailored structural attributes.

## 2. Polymer Matrix for Structural Wave-Absorbing Functional Integrated Composites

The resilience of composite materials to high temperatures and their capacity to bear mechanical loads are critical determinants of their versatile application. Materials endowed with exceptional wave-absorbing capabilities are consequently deployed in specialized sectors, including aeronautics, astronautics, and the military. Their exceptional thermal resistance qualifies them for deployment in extreme operational conditions, while their formidable mechanical properties guarantee their structural integrity under load. These dual attributes are also key performance indicators for structural wave-absorbing materials. The thermal resilience of fiber-reinforced wave-absorbing composites hinges on the selection of the resin matrix. The choice of reinforcement fibers and the resin matrix together dictate the composite’s mechanical performance. Augmenting the composite’s capabilities can be accomplished through a variety of approaches, such as the strategic combination of disparate resin matrices with reinforcement fibers, the modification of the resin matrix, surface treatments, the integration of additives, or the introduction of supplementary fillers.

### 2.1. Polyimide

Polyimides, distinguished by their imide ring-laden backbones, are crafted through a meticulous sequence of condensation reactions between tetracarboxylic dianhydrides and diamines [2]. The aromatic ring structures embedded in their main chains confer upon these polymers remarkable thermal stability and formidable resistance to shear forces [4]. The elevated activation energy for the spatial rotation of their molecular chains, alongside the covalent bonds’ inherent resistance to motion at high temperatures, endows PI materials with an impressive amorphous glass transition temperature and superior heat tolerance [5]. These intrinsic properties have positioned polyimides as exemplary performers in the realms of mechanical engineering, energy technology, and the innovative domain of advanced materials. Moreover, through strategic molecular-configuration optimization, polyimides can also manifest superior insulating characteristics, thereby expanding their utility across the spectrum of electrical device fabrication, electronic product assembly, and precision component manufacturing [6]. 

**Figure 1:**
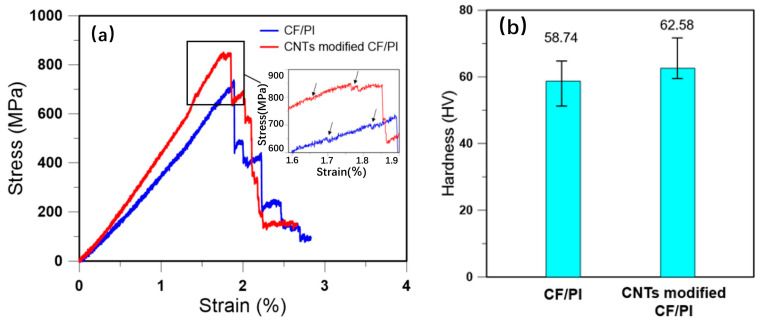
(**a**) Bending stress–strain curves of CF/PIs and CNT-modified CF/PIs [7]. Reproduced with permission from Yue Liu, *International Journal of Fatigue*; published by Elsevier, 2023. (**b**) Surface hardness of CF/PIs and CNT-modified CF/PIs [7]. Reproduced with permission from Yue Liu, *International Journal of Fatigue*; published by Elsevier, 2023.

Liu et al. [7] have enhanced carbon fibers (CFs) with fabric-reinforced PI composites (CF/PIs) by incorporating carbon nanotubes (CNTs) and investigated the bending performance of the CNT-modified CF/PIs at room temperature. As depicted in Figure 1a, both the pristine CF/PIs and the CNT-modified CF/PIs exhibit a linear increase in bending stress with increasing bending strain, followed by fluctuations near the ultimate bending stress. During this phase, the material does not fracture, indicating that the fibers continue to contribute to the mechanical load-bearing capacity despite the inhomogeneous stress distribution within the material. The average bending strength and modulus of the CF/PIs and CNT-modified CF/PIs are 746.91 MPa and 840.06 MPa, and 43.32 GPa and 49.48 GPa, respectively. Compared to the CF/PIs, the bending strength and modulus of the CNT-modified composites have been improved by 12.47% and 14.22%, respectively. These results substantiate the enhanced bending performance of the CF/PIs post-CNT modification. Furthermore, as shown in Figure 1b, the average surface hardness of the CF/PIs and CNT-modified CF/PIs are 58.74 HV and 62.58 HV, respectively, demonstrating that the dispersion of CNTs within the resin matrix also confers a hardness improvement to the CF/PIs. Song et al. [8] have ingeniously employed a biomimetic polydopamine (PDA) coating strategy to augment the interfacial synergy between the CF and the PI matrix, culminating in the development of PDA-CF/PI composites. The insulating nature of PI is characterized by a modest thermal conductivity of 0.17 W/(mK), which is substantially elevated to 0.49 W/(mK) upon the integration of CFs. Remarkably, the PDA-CF/PI composites demonstrate an even more pronounced thermal conductivity of 0.69 W/(mK), attributed to the enhanced interfacial interactions. The tensile properties of pristine PI are quantified with a tensile strength and modulus of 39.45 ± 6.3 MPa and 1.016 ± 0.05 GPa, respectively. In contrast, the PDA-CF/PI composites showcase a dramatic enhancement in tensile strength to 122.226 MPa and modulus to 16.314 ± 0.5 GPa. Comparatively, the CF/PIs exhibit a tensile strength and modulus of 105.68 MPa and 5.675 ± 0.5 GPa. The PDA-induced interfacial modification on CF surfaces has precipitated a significant escalation in tensile strength by 16.4% and an astounding 187.5% improvement in tensile modulus. Peng et al. [9] fabricated composite materials using CFs [10] as the reinforcing agent and PI as the matrix resin. By grafting modifications on the surface of the CF, the interfacial properties of the CF/PIs were enhanced. Experimental measurements revealed that as the oxygen content increased from 0 to 5%, the flexural modulus and strength of the PI composites improved significantly. Specifically, the flexural strength of the CF/PI material increased from 2475 MPa to 2780 MPa. Wang et al. [11] have delved into the effects of high-temperature thermal oxidation on the mechanical integrity of phenylethynyl-terminated PI (PETI) [12] reinforced with CFs (CFRC). This class of PI, renowned for its robust mechanical and structural attributes even at 371 °C, is synthesized from phenylethynyl-terminated imide oligomers, showcasing a superior processability ideal for composite fabrication. The study meticulously assessed the impact of prolonged thermal exposure on the mechanical characteristics of PETI/CF composites. The initial mechanical assessments of the composites yielded remarkable values for flexural strength, modulus, and interlaminar shear strength, standing at 1750.8 MPa, 165.8 GPa, and 102.46 MPa, respectively. Post-exposure to 6000 min of thermal oxidation at the critical temperature of 371 °C, the composites exhibited a commendable retention of flexural strength and interlaminar shear strength at 1342.5 MPa and 67.37 MPa, respectively. Interestingly, the flexural modulus escalated to 171.8 GPa, suggesting a potential enhancement in stiffness under oxidative conditions. The study by Wang et al. [11] provides compelling insights into the resilience of PETI/CF composites under severe thermal oxidative conditions, highlighting their potential for enduring applications in high-stress environments where conventional materials may falter.

### 2.2. Epoxy Resins

Epoxy resins, defined by their molecular architecture that incorporates two or more epoxy groups susceptible to ring-opening reactions, are frequently encountered as composites imbued with curing agents and a spectrum of additives, or as the cured end products. These polymers are distinguished by their exceptional reactivity and workability, coupled with their remarkable adhesion, mechanical strength, electrical properties, robust resistance to both acidic and alkaline environments, and economic efficiency [13].

**Figure 2:**
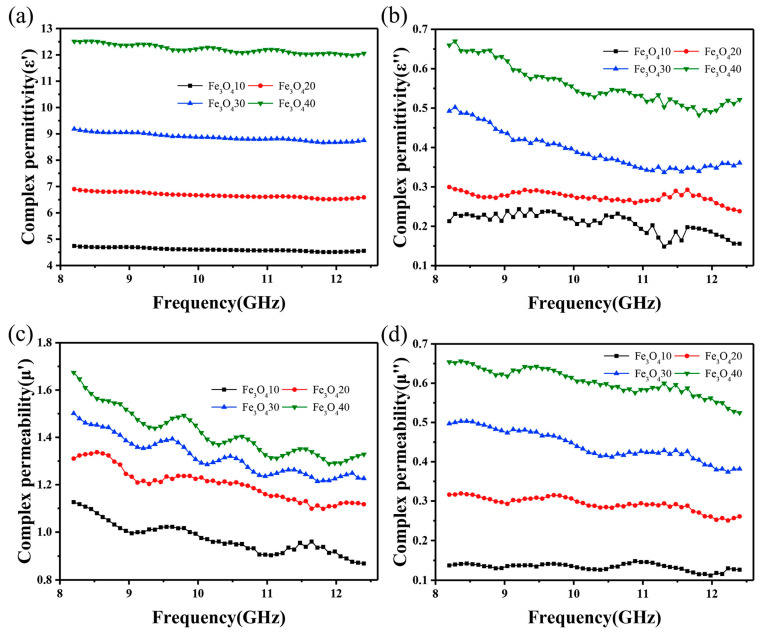
Frequency dependence of (**a**,**c**) real part, (**b**,**d**) imaginary part of complex permittivity and complex permeability of glass fiber/Fe_3_O_4_/epoxy composites [18]. Reproduced with permission from Wei Chen, *Polymer Testing*; published by Elsevier, 2020.

Shin et al. [14] have masterfully incorporated multi-walled carbon nanotubes (MWCNTs) into an epoxy matrix, crafting a glass-fiber composite prepreg with exceptional electromagnetic wave-absorption properties. The tensile and shear testing of the glass-fiber/MWCNT 1.8 wt%/epoxy composites has unveiled a tensile strength of 467 MPa and a tensile modulus of 29.1 GPa, while the shear strength and modulus stand at 47.9 MPa and 5.51 GPa, respectively. These mechanical properties are not only on par with the tensile modulus of commercially available glass-fiber epoxy composites but also exhibit a superior tensile strength. Zhang et al. [15] designed a composite material using epoxy resin as the matrix, glass fiber as the reinforcement, and alumina (Al_2_O_3_) as the thermally conductive filler. The manufacturing process included vacuum-assisted flow casting and hand-lay-up molding techniques to produce Al_2_O_3_-modified epoxy-resin fiberglass composites. The composites were analyzed by varying the content of Al_2_O_3_ in comparison with a control group without filler. The experimental results showed that when the Al_2_O_3_ content reached 2%, the thermal conductivity increased by about 20% and the specific heat capacity increased by about 30%. In addition, the burning time was extended by 10 s. Although the tensile and impact properties decreased, the hardness of the samples increased regardless of the fabrication process. It is noteworthy that the mechanical properties of the samples fabricated by vacuum-assisted flow casting were better than those fabricated by hand-lay-up molding, while the Al_2_O_3_ content was kept constant. Mahtar et al. [16] designed a composite material consisting of an epoxy-resin matrix reinforced by glass fibers and further reinforced by the addition of amine-functionalized graphene oxide (fGO). The fGO particles were sprayed onto the surface of the glass fibers using a spraying technique, and then the composite was fabricated by vacuum-assisted resin infusion. The tensile strength of the unmodified glass fiber-reinforced composites at 28 °C was 215.62 ± 13.38 MPa. The tensile strength increased linearly with increasing fGO content, with a slope of 22.98 MPa per weight percent. Specifically, the tensile strength of the composites increased by 17.6% at 1 wt% fGO content. Similar increases were observed for the tensile modulus and tensile breaking strain. In addition, the fatigue life of the fGO-modified glass fiber-reinforced epoxy composites subjected to maximum stresses of 124 and 150 MPa at 90 °C was increased by a factor of 8 and 30, respectively, as compared to the unmodified composites. Perumal et al. [17] incorporated zirconium silicate particles into an epoxy-resin matrix to enhance the mechanical properties of glass fiber-reinforced epoxy-resin composites. The tensile strength, tensile modulus, flexural strength and flexural modulus of the pristine glass fiber-reinforced epoxy composites were about 188 MPa, 37.5 GPa, 296 MPa and 23.6 GPa, respectively. With the addition of 10 wt% zirconium silicate particles, the mechanical properties of the composites were significantly improved compared to their unmodified counterparts. Specifically, the tensile strength, tensile modulus, flexural strength and flexural modulus increased by 33.33%, 12.56%, 32.88% and 15.74%, respectively. The tensile test showed an 82.96% increase in strain energy and a 53.52% increase in elongation at break. Similarly, a bending test showed a 52.38% increase in strain energy and a 14.47% increase in elongation at break. Chen et al. [18] have investigated the relationship between the reflection loss, complex permittivity, and complex permeability of glass-fiber/Fe_3_O_4_/epoxy-resin composites with varying concentrations of Fe_3_O_4_ nanoparticles. Their study reveals that composites with 10 wt% and 20 wt% Fe_3_O_4_ exhibit suboptimal microwave-absorption capabilities. However, at an Fe_3_O_4_ concentration of 30 wt% and a thickness of 2.2 mm, the composite achieved a minimum reflection loss of 15.9 dB with an effective absorption bandwidth of 3.5 GHz (8.2–11.7 GHz). Intriguingly, when the concentration of Fe_3_O_4_ is increased to 40 wt% and the thickness is reduced to 1.6 mm, the minimum reflection loss improves significantly to 43.5 dB, with an absorption bandwidth of 3.7 GHz (8.7–12.4 GHz). As shown in Figure 2a,b, the study’s graphical data illustrate that as the content of Fe_3_O_4_ nanoparticles escalates, both the real part (ε′) and the imaginary part (ε″) of the complex permittivity increase, with ε′ ranging from 4.7–4.5 to 12.5–12.1 and ε″ from 0.2–0.1 to 0.7–0.5, respectively. Correspondingly, as shown in Figure 2c,d, the real (μ′) and imaginary (μ″) parts of the complex permeability also increase, with μ′ enhancing from 1.1–0.9 to 1.7–1.3 and μ″ from 0.1–0.1 to 0.7–0.6 as the Fe_3_O_4_ concentration is elevated from 10 wt% to 40 wt%. This suggests that higher concentrations of Fe_3_O_4_ nanoparticles lead to the greater complex permeability of the composite material, which in turn facilitates better impedance matching and increased microwave-absorption capacity. The enhancement in the electromagnetic loss of the composite material can be attributed to the combined increase in dielectric and magnetic losses.

### 2.3. Other Resin Matrix

Dong et al. [19] enhanced the mechanical properties of polyphenylene sulfide (PPS)/CF composites by employing a carboxyl-functionalized sizing agent. They synthesized a low-molecular-weight carboxylic acid-terminated PPS and coated it onto plasma-treated CFs. The resulting composites exhibited a tensile strength of up to 115.20 MPa, substantiating the improvement in mechanical performance. Yu et al. [20] have developed an innovative CF/bismaleimid(BMI)composite material, enriched with Fe@C nanoparticles encapsulated within a phenolphthalein polyetherketone(PEK-C)nanofilm, and subjected it to interlaminar shear strength (ILSS) testing. The findings demonstrate a modest yet significant enhancement in ILSS, escalating from 105.1 MPa to 109.8 MPa—a 4.2% increase—as the concentration of Fe@C within the PEK-C nanofilm reached 20 wt%. This improvement is credited to the mechanical interlocking effect of the dispersed thermosetting BMI, which encapsulates the thermoplastic PEK-C phase, creating an interpenetrating network that bolsters the interlaminar shear properties. Beyond this, other matrix resins have demonstrated remarkable utility across diverse applications. Cyanate ester (CE) resins, for example, exhibit superior heat and moisture resistance, positioning them as ideal candidates for structural components in missiles and spacecraft. Benzoxazine (BX) resins, akin to phenolic resins, are celebrated for their low melt viscosity and their ability to impart low moisture absorption and shrinkage rates to composites [21,22]. Moreover, BX resins are noted for their exceptional flame retardancy and dielectric properties. Notably, cyanate resins (PN) are serviceable at extreme temperatures of up to 816 °C, aligning with the demanding requirements of composite components in missile applications, and they also showcase commendable compressive and shear strength characteristics.

## 3. Wave-Absorbing Agents for Structure-Absorption Functional Integration Composites

Absorbents, differentiated by their underlying mechanisms, are classified into two principal types: electrical loss and magnetic loss. Within the electrical-loss category, further distinctions are made between resistive- and dielectric-loss absorbents. Materials designed for electrical-loss absorption are characterized by elevated complex permittivities and substantial dielectric-loss tangents, facilitating the conversion of electromagnetic waves into alternative energy forms through interactions with the electric field within the medium. Magnetic-loss absorbents are identified by their significant magnetic-loss tangents, with absorption attributed to mechanisms such as eddy current losses, magnetic after-effects, and hysteresis losses that arise from the medium’s engagement with the electromagnetic field [23]. The primary constituents of magnetic-loss absorbents encompass ferrites, supermetal powders, and carbonyl iron, among others [24,25].

### 3.1. Electric Loss-Type Wave Absorber

Resistive-loss absorbent materials facilitate the transformation of electromagnetic waves into thermal energy through the medium’s interaction with the electric field, inducing the movement of charge carriers and generating current [23]. The efficacy of such absorbents hinges on their electrical conductivity, with carbon-based materials, including graphite, CFs, and CNTs, predominating in this category. Dielectric-loss absorbents, on the other hand, dissipate electromagnetic energy via medium polarization mechanisms, such as dipole and space charge polarization, with relaxation phenomena during polarization consuming the energy of the electromagnetic waves [26]. Silicon carbide [27], barium titanate [28], and titanium dioxide [29] are exemplars of this class of absorbents. Carbon-based absorbents are favored for their ease of fabrication, elevated electrical conductivity, reduced density, and dispersibility. However, they present challenges when utilized in isolation, as their permittivity markedly deviates from the impedance of free space, leading to impedance mismatch and suboptimal absorption efficacy. Gupta et al. [30] investigated the incorporation of carbon black (CB) into polyurethane to assess how varying mass fractions affect the composite’s absorption capabilities, finding that at a 6.55% mass fraction and 1.3 mm thickness, the reflective loss at 17 GHz can attain −31.39 dB. CNTs, valued for their distinctive structural, mechanical, and electrical attributes, are increasingly integrated into absorbent materials to ameliorate their absorption properties. Their diminutive size coupled with a high surface area augurs well for applications within the absorption domain. CNTs can be amalgamated with magnetic-loss materials to offset their low magnetic permeability. Zhang et al. [31] explored the absorption performance of a mixture of MWCNTs and Fe_2_O_3_ across a 2–18 GHz frequency band, noting a reflective loss of −35.8 dB at 8.6 GHz. CFs, recognized for their exceptional strength-to-weight ratio and electrical characteristics, have been leveraged by LIU et al. [32] to synthesize cobalt oxide/CF (CoO_x_-CF) composites. These composites demonstrated a reflective loss of −45.16 dB at a thickness of 1.5 mm, with a bandwidth of 13 GHz achieving reflective losses below the −10 dB threshold.

### 3.2. Magnetic Loss-Type Wave Absorbers

Magnetic-loss absorbents, such as ferrites, carbonyl iron powders, carbonyl nickel powders, and other metallic micro-powders, are renowned for their elevated magnetic permeability and magnetic-loss characteristics. Ferrites are categorized into spinel, garnet, and magnetoplumbite types based on their structural configurations. They exhibit both magnetic and electrical losses. Mušič et al. [33] have meticulously developed a composite material based on an organic resin solution, engineered for the purpose of electromagnetic absorption and infused with magnetic fillers derived from the Mn_0.66_Zn_0.27_Fe_2.07_O_4_ system. The study’s focus was to elucidate the electromagnetic absorption characteristics of the composite material when the weight ratio of ferrite powder to acrylic resin polymer solution was finely tuned to 75:25. The ferrite powders, processed through three distinct techniques, consistently demonstrated absorption peak values with a reflection loss of less than −10 dB within a 5 mm composite layer. This discovery accentuates the pivotal role of composite layer thickness in determining the material’s absorption efficiency—a critical parameter in the advancement of sophisticated electromagnetic shielding materials. Qing et al. [34] developed a coating with flaky carbonyl iron powder as the absorbent and an epoxy–silicone resin matrix. This coating, with 55 wt% carbonyl iron content, reached a reflection loss of −42.5 dB at 10.6 GHz. Moreover, at a material thickness of 2 mm, the frequency bandwidth with a reflection loss below −5 dB for any composite content exceeded 10 GHz, underscoring the coating’s superior microwave-absorption capabilities.

### 3.3. Composite Wave Absorber

**Figure 3:**
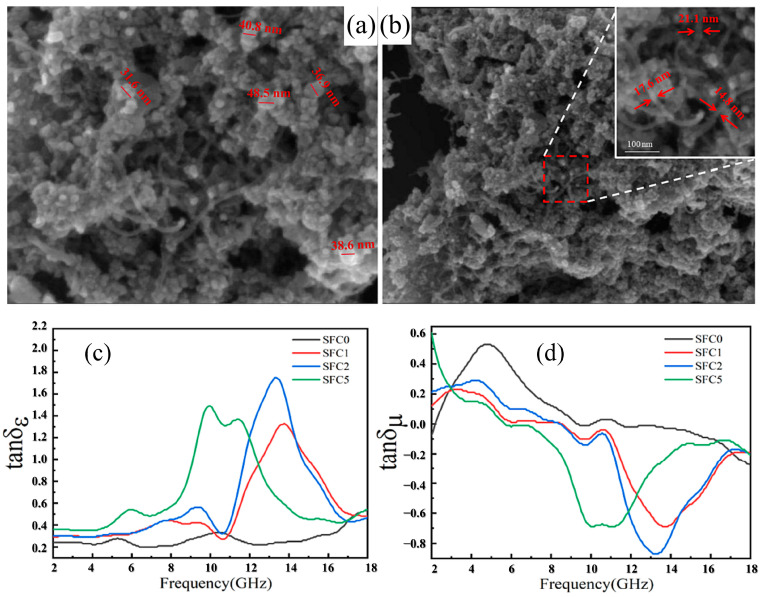
(**a**,**b**) SEM images of Sr-doped Fe_3_O_4_/CNTs nanocomposites (1 wt% CNTs) [40]. Reproduced with permission from W.Q. Guo, *Diamond and Related Materials;* published by Elsevier, 2024. (**c**) Dielectric-loss tangent and (**d**) magnetic-loss tangent of Sr-doped Fe_3_O_4_/CNTs nanocomposites [40]. Reproduced with permission from W.Q. Guo, *Diamond and Related Materials*; published by Elsevier, 2024.

Incorporating a solitary absorbent species into composite materials seldom yields the optimal microwave-absorption characteristics desired. The synergistic incorporation of dual electrical loss-type absorbents within the resin matrix can significantly refine electromagnetic properties, thus augmenting the composite’s absorption efficacy. Prevalent hybrid approaches include the amalgamation of CB with MWCNTs [35], the combination of CB with silicon carbide (SiC) nanoparticles [27], and the union of CB with acicular zinc oxide (ZnO) [36]. Li et al. [37] have pioneered the synthesis of a novel composite material, seamlessly amalgamating graphene, CNTs, tetrairon trioxide, and polydimethylsiloxane, employing an electrophoretic self-assembly methodology. The absorption prowess of this composite has been rigorously investigated, revealing that at a thickness of 1.42 mm, it can attain a profound minimum reflection loss of −50.5 dB. Moreover, the material exhibits an extraordinary bandwidth, with reflection losses consistently dipping below the −10 dB threshold, extending across a remarkable 5.7 GHz spectrum. In another study, the electromagnetic properties of nickel-coated short CFs were notably enhanced with the integration of MWCNTs, as investigated by Rosa et al. [38] within the 8–18 GHz frequency spectrum. By fusing electrical-loss absorbents with magnetic-loss counterparts, a dual absorption mechanism is harnessed to escalate the composite’s absorption capabilities. Qing et al. [39] fine-tuned the ratio of carbonyl iron particles to graphene flakes within their composite, and at a material thickness of 0.9 mm, the bandwidth with reflection losses beneath −10 dB extended to 12 GHz, signifying a substantial enhancement in absorption bandwidth. Guo et al. [40] have fabricated a composite absorber material through a hydrothermal synthesis that integrates CNTs with strontium (Sr)-doped (Fe_3_O_4_) nanoparticles. The average diameter of the Sr-doped Fe_3_O_4_ nanoparticles, as measured in Figure 3a, is less than 40 nm. Figure 3b distinctly portrays CNTs with diameters ranging from 15 to 20 nm, uniformly interspersed within the Sr-doped Fe_3_O_4_/CNTs nanocomposite, creating a stable three-dimensional network structure. Furthermore, the smaller nanoparticles are observed to aggregate due to dipole interactions and the excellent magnetic properties of the nanoparticles, leading to the formation of effective interfaces between CNTs and Fe_3_O_4_ nanoparticles. Consequently, the electromagnetic loss of the composite absorber material is not merely a summation of the resistive loss of CNTs and the magnetic loss of Fe_3_O_4_ but also encompasses losses due to interfacial polarization, including polarization at the interfaces between Fe_3_O_4_ nanoparticles and between CNTs and Fe_3_O_4_. The dielectric-loss tangent (tanδ_ϵ_) and the magnetic-loss tangent (tanδ_μ_) are instrumental in delineating the absorptive capacities of dielectric and magnetic components within microwave composites. In the Sr-doped Fe_3_O_4_/CNTs nanocomposite, the representation of tanδ_ϵ_ and tanδ_μ_ in Figure 3c,d, respectively, unveils a transition in loss mechanisms across varying frequencies. Depending on the CNT percentages of 0 wt%, 1 wt%, 2 wt% and 5 wt% in the initial solution, Sr-doped Fe_3_O_4_/CNTs nanocomposites are labeled SFC0, SFC1, SFC2 and SFC5, respectively. Initially, in the low-frequency regime (below 8 GHz), magnetic losses predominate for the SFC0 composite. This is followed by a crossover to dielectric loss and culminates in a synergistic contribution from both dielectric and magnetic losses in the high-frequency domain (beyond 16 GHz). The Sr-doped variants, SFC1, SFC2, and SFC5, exhibit a distinct behavior, with the incorporation of CNTs intensifying tanδ_ϵ_ and attenuating tanδ_μ_. Peaks in tanδ_ϵ_ for these composites are observed at 10 GHz, 13.1 GHz, and 14 GHz, with values reaching 1.4, 1.7, and 1.3, respectively. This underscores the interplay between dielectric and magnetic losses in dictating the microwave-absorption efficacy of the Sr-doped Fe_3_O_4_/CNTs nanocomposites. The experimental results for SCF1, with a thickness of 3 mm, reveal a remarkable minimum reflection loss at 6.32 GHz, reaching an impressive −65.54 dB.

## 4. Wave-Absorbing Mechanisms in Structure-Absorption Functionally Integrated Composites

### 4.1. Electromagnetic Shielding Mechanism

**Figure 4:**
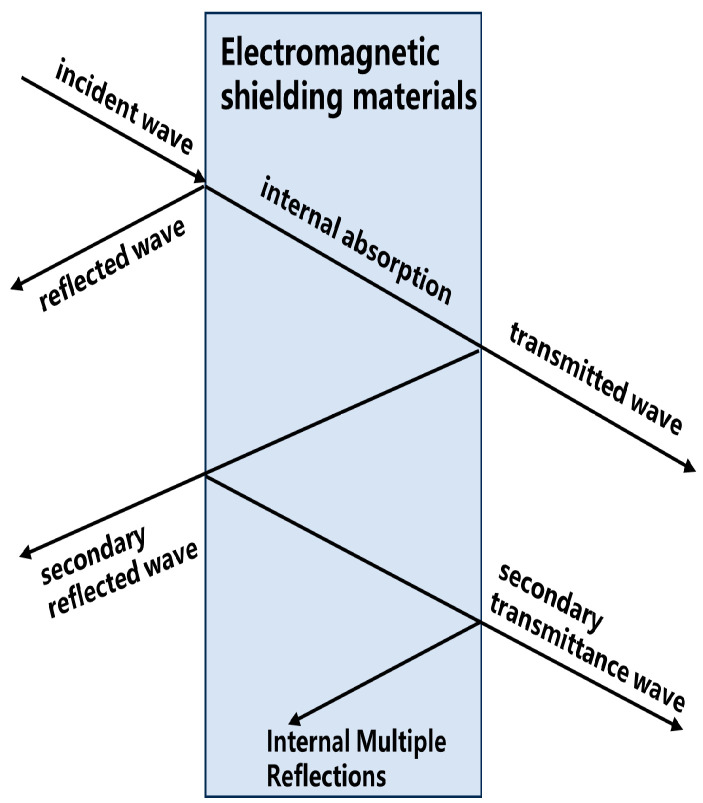
Electromagnetic shielding mechanism diagram.

Upon encountering a material, electromagnetic waves are subject to a triad of outcomes: as shown in Figure 4, a segment is reflected at the material’s surface, another fraction proceeds into the material’s interior where it experiences successive reflections that may nullify each other or is engorged by the absorbent material, and a residual portion traverses the material, continuing in transmission. The role of electromagnetic shielding is to impede the transmission of these waves—a feat typically accomplished through reflective or absorptive processes. The mechanism of action of electromagnetic wave-absorbing materials primarily involves their capability to efficiently convert incident electromagnetic wave energy into thermal energy or other forms of energy dissipation, thereby reducing or eliminating the reflection of electromagnetic waves.

### 4.2. Wave-Absorption Conditions and Principles

For efficacious absorption, absorbent materials are subject to two critical conditions. Firstly, impedance matching is essential to curtail the reflection of electromagnetic waves at the surface, ensuring their unimpeded ingress into the material. Secondly, attenuation must be maximized, guaranteeing that the waves, once inside the material, are entirely consumed and attenuated. The realization of both impedance matching and electromagnetic wave attenuation hinges on the material’s electromagnetic characteristics, encapsulated by the complex permittivity and permeability, as articulated in Equations (1) and (2) [41].

(1) $$\varepsilon_{r}=\varepsilon_{r}^{\prime }-j\varepsilon_{r}^{\prime \prime }$$

(2) $$\mu_{r}=\mu_{r}^{\prime }-j\mu_{r}^{\prime \prime }$$

where $\varepsilon_{r}$ is the complex permittivity; $\mu_{r}$ is the complex permeability; $\varepsilon_{r}^{\prime }$ is the real part of the complex dielectric constant; $\varepsilon_{r}^{\prime \prime }$ is the imaginary part of the complex dielectric constant; $\mu_{r}^{\prime }$ is the real part of the complex permeability; and $\mu_{r}^{\prime \prime }$ is the imaginary part of the complex permeability

In the realm of engineering, the loss tangent, namely the dielectric-loss tangent delineated by Equation (3) and the magnetic-loss tangent delineated by Equation (4), serves as a standard metric for assessing the dissipative attributes of materials. Absorbent materials with more substantial imaginary portions of the permittivity and permeability can induce a greater attenuation of the incident electromagnetic waves, thereby enhancing the material’s absorptive capabilities [42].

(3) $$\mathrm{Tan}\delta \epsilon =\frac{\varepsilon^{\prime \prime }}{\varepsilon^{\prime }}$$

(4) $$\mathrm{Tan}\delta \mu =\frac{\mu^{\prime \prime }}{\mu^{\prime }}$$

The performance of wave-absorbing materials can be measured by the absorbance *A*(*ω*), which is related to the reflectance *R*(*ω*) and transmittance *T*(*ω*), as in Equation (5).

(5) $$A\left(\omega \right)=1-R\left(\omega \right)-T\left(\omega \right)$$

(6) $$Z_{1}=\sqrt{\frac{\mu_{1}}{\varepsilon_{1}}}=\sqrt{\frac{\mu_{0}}{\varepsilon_{0}}}=Z_{0}$$

Consequently, the strategic design of a material’s elemental structure to align its equivalent electromagnetic parameters—permittivity and permeability—reaches an optimal state where the material’s wave impedance, $Z_{1}$, matches that of free space, $Z_{0}$. This impedance matching facilitates the unobstructed entry of the incident wave into the absorbent medium, with no reflection occurring. The energy dissipation is driven by the imaginary components of these complex parameters. Consequently, by meticulously engineering the imaginary parts, one can maximize energy dissipation, curtailing transmission and thereby attaining highly efficient absorption [43].

The performance of wave-absorbing materials can also be measured by substituting the electromagnetic parameters of the material into Equations (7) and (8) to calculate the input impedance and reflection loss of the wave-absorbing material based on the transmission line theory [18].

(7) $$Z_{in}=Z_{0}\sqrt{\frac{\mu_{r}}{\varepsilon_{r}}}\tan[j(\frac{2\pi fd}{c})\sqrt{\varepsilon_{r}\mu_{r}}]$$

(8) $$R_{L}=20lg|\frac{Z_{in}-Z_{0}}{Z_{in}+Z_{0}}|$$

where $Z_{in}$ is the input characteristic impedance, Ω; $Z_{0}$ is the free space impedance, Ω; *F* is the electromagnetic frequency, GHz; *d* is the material thickness, mm; *C* is the speed of light, m/s; and $R_{L}$ is the reflection loss, dB. 

The proficiency of an absorbent material is inversely proportional to the magnitude of its reflection loss, with lower values denoting enhanced absorption efficacy. Materials that exhibit reflection losses below −10 dB across an expansive bandwidth are considered to possess superior absorbing properties. The attainment of a −10 dB reflection loss threshold is a critical milestone, indicating that 90% of the incident electromagnetic waves have been effectively absorbed, thereby reflecting the material’s excellence in electromagnetic wave absorption.

## 5. Methods of Constructing Structural Wave-Absorbing Functionally Integrated Composites

The initial application of microwave-absorbent materials took the form of coatings, which, due to their straightforward application process, convenience, and adjustable parameters, garnered global attention and found extensive utility in military armaments. Stealth weapon systems universally integrate these coating-type absorbents; for example, the American F-117 Nighthawk and B-2 Spirit employ such coatings to achieve stealth capabilities. Liu et al. [24] demonstrated that an optimal absorbent performance is achieved with a mixture containing 20% Ni and 80% Sr-W-type ferrite, showcasing the lowest reflection rate and an absorption peak at −20.69 dB at the frequency of 12.08 GHz, with a bandwidth below −10 dB spanning 4 GHz. Their investigation into the thickness of the absorbent coating revealed that an increase in thickness shifts the absorption peak to lower frequencies, and at a certain thickness, the material exhibits a dual absorption peak within the 2 to 18 GHz range. Xia et al. [44] layered CNTs with epoxy resin on 1–2 mm aluminum plates in an 8:100 ratio, observing a dual peak absorption at a composite coating thickness of 7 mm, with the primary peak reflecting at −21.10 dB at 10.08 GHz and a secondary peak at −20.20 dB at the same frequency, achieving a broad bandwidth with reflection losses below 8 dB of 5.46 GHz. Wang et al. [45] crafted a ferrite/epoxy composite coating and evaluated the impact of varying weight ratios of ferrite to polymer on absorption efficacy. Their findings indicated that a 3:20 mass ratio of ferrite to epoxy resin could achieve a reflection loss of −16 dB at 800 MHz, with losses below −10 dB maintained across the 700 MHz to 1 GHz range.

Despite the simplicity and convenience of the manufacturing process of coating-type absorbent materials, their application scope is circumscribed by several limitations, including high density, a restricted absorption frequency band, inadequate bonding and stability with the substrate, a propensity for aging and delamination, and suboptimal efficiency. As technology has progressed, there has been a burgeoning interest in the study of structural absorbent materials. These materials achieve a harmonious integration of structure and function, employing a resin matrix and fibers for reinforcement while incorporating absorbents to ensure the material’s wave-absorbing properties. Furthermore, the absorption performance can be optimized through thoughtful structural design, which also endows the material with commendable mechanical strength, thermal stability, and resistance to oxidation. The subsequent discussion will elucidate structural absorbent materials, exploring configurations such as single-layer, multi-layer, and multi-layer sandwich structures.

### 5.1. Single-Layer Construction

The distinctive feature of a single-layer structure lies in its ability to modulate the electromagnetic absorption characteristics by simply altering the type and concentration of the absorbent agents, as well as the thickness of the material. Ren et al. [46] prepared an absorption composite with epoxy cyanate ester (EP-CE) as the matrix and graphene nanosheets (GNSs) as the absorbent. They studied its absorption performance and found that at a GNS content of 3% and a material thickness of 4 mm, the composite could achieve a loss of −21.4 dB at 4.5 GHz, with reflection losses consistently below −10 dB within the 4 to 4.9 GHz frequency band. Wang et al. [47] have ingeniously utilized MWCNTs as fillers within a PI matrix to fabricate composite materials. Their investigation delved into the influence of varying MWCNT concentrations on the electromagnetic absorption properties of these composites. Notably, at MWCNT loadings of 3% and 4%, the reflection loss values, which are indicative of absorption performance, dipped below the −5 dB threshold across effective bandwidths of 3.57 GHz and 3.67 GHz, respectively. Intriguingly, when the MWCNT content was elevated to 5%, the reflection loss consistently remained below −5 dB across the entire measured frequency range, with a bandwidth of 1.5 GHz where the loss was even more pronounced, dropping below −10 dB. These findings underscore the significant impact that the type and concentration of the absorbent fillers have on the absorption characteristics of the composite material. Zhao et al. [48] chose Fe-Ni alloy powder as a nanofiller material and incorporated it into an epoxy-resin matrix to make composites that can be used as microwave-absorbing interlayers. The electromagnetic wave-absorption properties of these single-layer nanocomposites were investigated meticulously. The experimental results show that when the thickness of the Fe-Ni alloy/epoxy-resin composite layer is precisely 1.5 mm, its absorption bandwidth reaches 3.73 GHz in the frequency range of 1–18 GHz, and the reflection loss is always lower than −10 dB. 

### 5.2. Multi-Story Structures

While single-layer structures offer the simplicity of adjusting electromagnetic absorption properties, they are not without their limitations. These include poor frequency-bandwidth tunability, limited scope for enhancing absorption performance, and inferior mechanical properties [49]. Chen et al. [18] have elegantly engineered a composite material with a meticulously designed matching layer, consisting of 0.5% MWCNTs interspersed within a glass-fiber epoxy-resin matrix. This is complemented by an absorption layer rich in 40% magnetite (Fe_3_O_4_) nanoparticles, also embedded in a glass-fiber epoxy resin. When subjected to absorption testing with the total thickness of the matching and absorption layers precisely adjusted to 1.8 mm, the composite material demonstrated a remarkable maximum reflection loss of −45.6 dB. Eun et al. [50] designed a composite material with a surface layer of glass fiber-reinforced epoxy resin and a multilayer absorbent core consisting of CNTs as the absorbent, glass fibers as the reinforcing agent, and epoxy resin as the matrix. They investigated the impact of layering on the absorption performance of the composite by constructing the core with three, four, and seven layers of CNTs/glass fiber/epoxy-resin composites. Experimental results showed that the unlayered composite achieved a reflection loss of up to −61.9 dB at 10 GHz. Kim et al. [51] developed a bilayer absorbent material composed of a ferrite and CF composite laminate, with a CF polymer composite serving as the reflective substrate and a ferrite/epoxy-resin composite as the absorbent layer. The experimental outcomes demonstrated that the bilayer composite exhibited superior absorption performance compared to a single layer of ferrite/epoxy-resin composite. As the thickness of the ferrite layer increased, the peak absorption shifted towards lower frequencies. With a thickness of 5 mm, the reflection loss at 7 GHz reached −37.6 dB. Geeri et al. [52] successfully fabricated composites with excellent electromagnetic absorption properties by incorporating MWCNTs as fillers into glass fiber-reinforced epoxy matrices. When these composites are synergized with pristine electrically conductive polymeric materials (PECs), they can exhibit excellent microwave-absorption properties. Notably, the PEC-coated composites exhibit a minimum reflection loss of −38.69 dB at a filler concentration of 5%, which is a significant improvement over the uncoated composites, which exhibit a minimum reflection loss of −9.4 dB.

### 5.3. Multi-Layer Sandwich Construction

In the realm of electromagnetic wave absorption, sandwich structures stand out for their effectiveness. By integrating a core material between two facing layers, these structures capitalize on the multiple scattering of electromagnetic waves, which, upon entering the structure, are subjected to a series of reflections and refractions. This intricate interaction within the core results in an enhanced interference absorption, thereby optimizing the overall absorption performance of the material. The sandwich structure’s design not only contributes to the material’s lightweight and robust attributes but also plays a pivotal role in its electromagnetic absorption capabilities, making it an ideal choice for applications where both structural integrity and absorption efficiency are paramount [53].

#### 5.3.1. Honeycomb Sandwich Construction

Honeycomb sandwich structures, as quintessential structural-type microwave absorbers, have been widely implemented and have demonstrated superior performance. Comprising a wave-permeable skin layer and an absorbent honeycomb layer, these layers are bonded and cured through an adhesive film [54]. The honeycomb layer, mimicking the hexagonal structure of natural beehives, is favored for its lightweight nature, coupled with considerable strength and rigidity, making it an ideal core in sandwich structures. The low density of honeycomb sandwich structures significantly reduces the overall weight of the system [55]. Moreover, the absorption performance can be optimized by tuning the geometric parameters of the honeycomb structure. 

**Figure 5:**
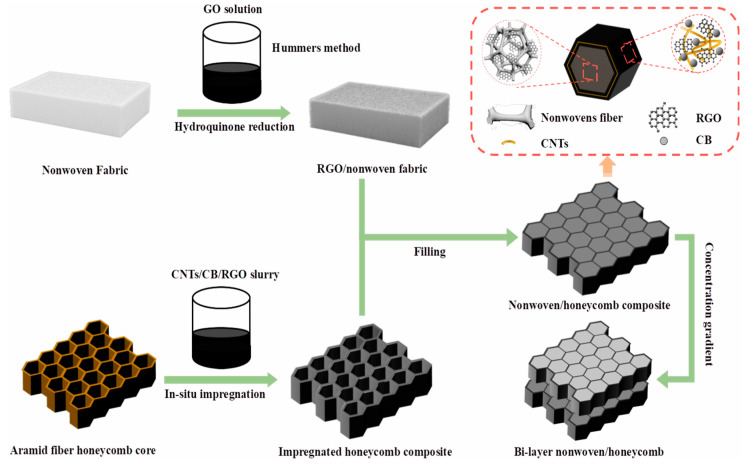
Schematic of preparation procedures of nonwoven/honeycomb composites [56]. Reproduced with permission from Hao Li, *Carbon*; published by Elsevier, 2024.

Li et al. [56] have developed an advanced composite material, integrating reduced graphene oxide (RGO) with a non-woven fabric to form a highly efficient electromagnetic wave absorber, ingeniously embedded within a honeycomb structure. Figure 5 illustrates the meticulous process of fabricating this RGO/non-woven fabric composite for the honeycomb structure. The non-woven fabric is initially immersed in a solution of oxidized graphene, followed by a series of advanced post-treatments to obtain the RGO/non-woven fabric composite. The aramid-fiber honeycomb core is then infused with a slurry containing CNTs, CB, and RGO, which is cured to yield a honeycomb structure with a robust surface coating. The final fabrication step involves the precise cutting of the RGO/non-woven fabric into hexagonal columns that replicate the internal geometry of the honeycomb. These are manually inserted into the honeycomb, creating the RGO/non-woven fabric honeycomb composite. Rigorous absorption testing has been conducted on this composite, revealing that at an oxidized graphene concentration of 5 mg/mL, with a mass ratio of CNTs and CB to RGO in the coating slurry of 2:1, and a single honeycomb layer thickness of 5 mm, the composite achieves a minimum reflection loss of −37 dB. Moreover, the bandwidth for which the reflection loss is below −10 dB is remarkably broad, spanning 12.1 GHz from 5.9 GHz to 18 GHz. Wu et al. [57] explored the impact of various structural parameters on the absorption characteristics of honeycomb structures. Experimental measurements reveal a significant trend in the acoustic properties of honeycomb structures: as the stature of these constructs escalates, their resonant frequencies exhibit a progressive diminution. Specifically, the structures, defined by a cell edge length of 3 mm and a cellular wall thickness of 0.2 mm, display a wall conductivity of 15 Siemens per meter. At a height of 6 mm, the reflection loss remains below −20 dB within the 13 GHz to 18.8 GHz frequency band, reaching a peak of −41 dB at 15.6 GHz. According to the experimental results, when the honeycomb structure side length is 3 mm, the honeycomb structure height is 6 mm, the honeycomb wall conductivity is 15 S/m, and the honeycomb wall thickness is 0.2 mm, the absorption effect is the best. Studying the effect of the edge length on the absorption performance, the results show that when the honeycomb wall thickness is 0.2 mm, the height of the honeycomb structure is 6 mm, the conductivity of the honeycomb wall is 15 S/m, and the honeycomb structure edge length is 3 mm, the reflection-loss effect is the best. Finally, the electrical conductivity of the honeycomb wall is analyzed, and for a given thickness of the honeycomb wall (0.2 mm), height of the honeycomb structure (6 mm), and side length of the honeycomb structure (3 mm), the optimum conductivity is 15 S/m. Therefore, the optimum parameters of the honeycomb structure are found to be a side length of 3 mm, a wall thickness of 0.2 mm, a height of 6 mm, and a conductivity of the honeycomb wall of 15 S/m. Wang et al. [58] have engineered a novel metasurface–honeycomb composite absorber material with a tri-layered structure. The design features a metasurface at the top, a honeycomb core, and a copper-clad base. An impedance-matching layer is strategically integrated into the honeycomb’s uppermost layer to augment the low-frequency absorption efficacy. The honeycomb is crafted from a phenolic resin-impregnated aramid paper laminate, and its surface is coated with a carbon layer designed for absorption. This honeycomb boasts exceptional properties such as being ultra-lightweight and high strength, and it has favorable dielectric characteristics. The carbon coating, in addition to its absorption capabilities, ensures that the composite material retains robust mechanical integrity. Empirical tests have confirmed that the absorber exhibits a reflectance loss consistently below −10 dB within the 3.65 to 17.3 GHz band, substantiating its wideband absorption proficiency. Choi et al. [59] proposed a honeycomb structure for microwave absorption, demonstrating its feasibility by utilizing the transverse nature of the honeycomb to absorb microwaves holistically. The absorbent honeycomb structure was fabricated using glass-fiber/epoxy MWCNT prepregs and an autoclave process. The absorption test results showed that the fabricated honeycomb structure achieved a reflection loss below −10 dB across the 3–16 GHz frequency band, with a peak of −35 dB at 7.7 GHz.

#### 5.3.2. Foam Sandwich Construction

The fabrication of foam-core materials typically involves blending thermoplastic or thermosetting resins with absorbents to form a resin matrix [60,61,62]. This matrix is then coated onto fiber surfaces to create prepreg, which is subsequently combined with a foam core through co-curing to form composite materials with electromagnetic absorption capabilities. Some methods also employ prepolymers mixed with absorbents to prepare the matrix material, followed by a polymerization foaming process. Foam-core composite materials not only broaden the absorption bandwidth of the materials but also offer the benefits of being lightweight, having high-temperature resistance, and corrosion resistance. Their unique foaming characteristics also facilitate the easy shaping of complex geometries. 

**Figure 6:**
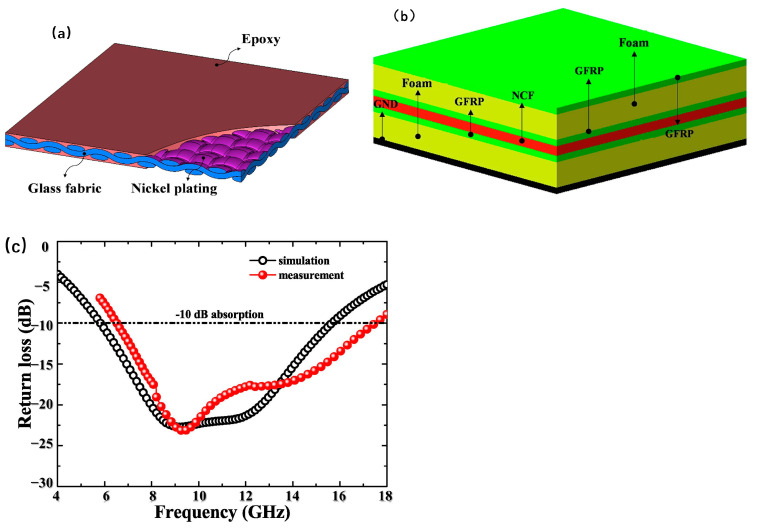
(**a**) Model of the nickel-coated fabric (NCF) radar-absorbing material, consisting of glass fabric, nickel plating, and epoxy [63]. Reproduced with permission from Won-Ho Choi, *Composite Structures;* published by Elsevier, 2020. (**b**) Foam sandwich wave-absorbing composites with NCF sandwich type [63]. Reproduced with permission from Won-Ho Choi, *Composite Structures;* published by Elsevier, 2020. (**c**) Comparison of wave-absorbing test results and simulation results of foam-cored wave-absorbing composites with NCF interlayers [63]. Reproduced with permission from Won-Ho Choi, *Composite Structures*; published by Elsevier, 2020.

Choi et al. [63] have introduced a radar-absorbing foam sandwich structure (RAS), as depicted in Figure 6b, which integrates a foam core with a glass fiber-reinforced polymer (GFRP) and chemically plated nickel-coated fabric (NCF). As shown in Figure 6a, the NCF is a composite of glass fabric, a nickel-plating layer, and epoxy resin. Sandwiched between the NCF layers, the foam core is strategically positioned to modulate impedance and broaden the absorption bandwidth without a significant increase in weight. The GFRP, which is interspersed between the foam core and NCF, serves to enhance the mechanical properties of the structure. The experimental results, illustrated in Figure 6c, demonstrate a reflection loss of up to −24 dB at approximately 9.3 GHz, with the reflection loss remaining below −10 dB across a frequency range of 6.4 to 17.4 GHz. Park et al. [64] have developed a composite material with superior absorption characteristics in the X-band frequency range (8.2–12.4 GHz), utilizing a sandwich structure. They infused the composite prepregs and polyurethane foam with conductive materials such as CB and MWCNTs to enhance the absorption performance. To bolster the mechanical properties of the composite, a structure was employed that integrates composite panels with a foam core. The face layers were fabricated from glass-fiber/epoxy-resin materials filled with CB and CF/epoxy-resin composites, with the core layer consisting of polyurethane foam containing MWCNTs. The composites were assembled into three, four, and five layers, adhering to the principle of using the CF/epoxy composite as the base layer and the foam core as the intermediate layer. The absorption performance was then rigorously tested. The experimental results demonstrated that the three-layer composite exhibited a reflection loss of less than −10 dB in the 9.1–11.1 GHz range. The four-layer composite showed a reflection loss below −10 dB in the 9.6–10.8 GHz range, and the five-layer composite achieved a reflection loss of less than −10 dB across the 8.7–11.7 GHz range. Wang et al. [65] have crafted a multilayer sandwich-structured composite material that integrates glass fiber-reinforced epoxy-resin composites, polyvinyl chloride (PVC) foam, CF-reinforced epoxy-resin composites, and frequency-selective surfaces. This innovative material not only exhibits excellent electromagnetic wave-absorption capabilities but also boasts superior mechanical load-bearing properties. The experimental testing has illuminated that this sandwich structure achieves a deep band of −15 dB, with a remarkable bandwidth spanning up to 14 GHz. As the thickness of the composite material increases, the structure is capable of maintaining a reflection loss below −10 dB across the 2 GHz to 18 GHz range, expanding the bandwidth to approximately 16 GHz.

#### 5.3.3. Other Sandwich Constructions

In addition to the conventional sandwich structures, there are other types such as corrugated core structures, lattice cores [66], and some special sandwich structures. The corrugated panel structure is a frequently utilized sandwich configuration, favored for its excellent mechanical properties characteristic of sandwich structures, such as being lightweight and high strength. Moreover, the layered distribution of the panel and the core, coupled with the hollow nature of the sandwich structure, facilitates the stratified arrangement of wave-transmitting, absorbing, and reflecting layers. This arrangement is conducive to the efficient absorption of electromagnetic waves and the attenuation or decay of the incident electromagnetic waves. When electromagnetic waves penetrate through the wave-transmitting layer into the absorption layer, they have a large contact area with the corrugations, which is beneficial for the dissipation of the waves [67]. Lattice-core sandwich structures adjust the absorption properties of materials by varying the shapes and arrangements of the lattices, often combined with foam cores. Beyond these sandwich structures, there are special configurations, such as the novel three-layer composite designed by Choi et al. [68], which consists of MWCNTs dispersed within a composite of epoxy resin and glass fiber, with a middle layer of epoxy resin and a cotton-fabric resistance layer. The study results indicate that this structure achieves a reflection loss of less than −10 dB within the 4.7 to 13.6 GHz frequency range.

### 5.4. Frequency Selection Surface

**Figure 7:**
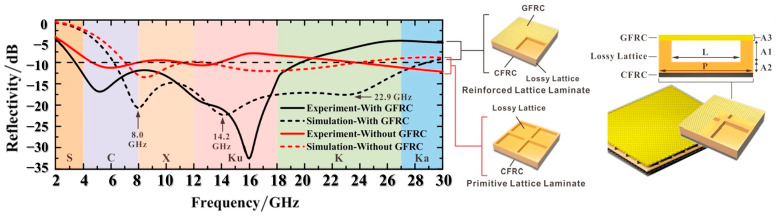
Experimental and simulated reflectivities of CF-enhanced lossy lattice structures with and without GFRC in the S (2 GHz–4 GHz), C (4 GHz–8 GHz), X (8 GHz–12 GHz), Ku (12 GHz–18 GHz), K (18 GHz–26.5 GHz), and Ka (26.5 GHz–30 GHz) bands. Three particular frequency points of the simulated absorption peaks, including 8.0 GHz, 14.2 GHz, and 22.9 GHz, are indicated in the figure. The right inset shows the schematic diagrams of the lattice structures with and without GFRC and the detailed designs and dimensions of the lossy lattice structures with GFRC and CFRC [71]. Reproduced with permission from Yixing Huang, *Carbon*; published by Elsevier, 2019.

Frequency-selective surfaces (FSS) are periodic structures arranged on the surface of a medium, capable of selectively transmitting electromagnetic waves based on varying angles of incidence and polarization states, effectively acting as a “filter” [69]. The performance of composite materials is influenced by numerous factors related to the FSS, including its position within the material, the shape and size of the periodic pattern, and more. Compared to traditional absorbents that enhance the absorption performance of composite materials, where the content of the absorbent affects the material thickness and the uniform dispersion of the absorbent within the matrix can impact the absorption performance, FSS-based absorbent materials can significantly improve the dispersion of the absorbent. Further in-depth research is warranted on FSS-based absorbent materials. Zhang et al. [31] designed and fabricated a CF/epoxy composite material with a grid structure and investigated the impact of grid position and the number of grid layers on absorption performance. The analysis revealed that composite materials with grids placed on the surface (grid unit size of 13 mm, matrix thickness of 4 mm, and CF K-number of 12 K) performed better than those with grids in the middle or at the bottom, achieving a reflection rate of −23.12 dB at 14.35 GHz. Moreover, the three-layer grid structure of the CF/epoxy composite material exhibited a reflection rate below −10 dB within the 10–18 GHz frequency range. Lee et al. [70] have developed a novel approach to fabricate a periodically patterned sheet through printed electronics, integrating a low-dielectric foam as the core material. The patterned layer was meticulously crafted on a PI-coated glass substrate, utilizing a low-conductivity paste formulated with graphite powder in polyurethane. Subsequent to the patterned layer’s development, a low-dielectric foam core layer was ingeniously designed. A copper sheet was strategically applied to the reverse side of the foam core, effectively shielding against electromagnetic wave penetration. The absorption performance, as evaluated through testing, demonstrated a significant reflection loss of approximately −21 dB at the frequency of 13.5 GHz. Remarkably, the reflection loss remained below the threshold of −10 dB across a substantial portion of the Ku band spectrum, spanning from 12.4 GHz to 18 GHz. Huang et al. [71] designed a novel CF/glass fiber-reinforced lossy-grid structure to enhance microwave-absorption performance and mechanical load-bearing capabilities. As illustrated, the glass fiber-reinforced composite material (GFRC) is located at the ribs of the grid, the CF-reinforced composite material (CFRC) is at the bottom of the grid, and the grid material consists of magnetic carbonyl iron particles and conductive MWCNTs embedded in an epoxy matrix. The optimization of the lossy lattice was ultimately determined with dimensions of L = 17.5 mm, P = 20 mm, A1 = 2 mm, A2 = 1 mm, A3 = 0.5 mm, and a total thickness of only 3.55 mm without CFRP as the base. As shown in Figure 7, the reflection loss with and without GFRC indicates that the structure without CFRP as the base exhibits two main absorption peaks at 16 GHz (−34 dB) and 5 GHz (−17.5 dB) within the 2 GHz to 18 GHz frequency band, achieving an absorption effect below −10 dB with a bandwidth of 16.31 GHz.

## 6. Conclusions and Outlook

In recent years, there has been an increasing demand for the integration of structural and functional properties in absorbent materials, prompting a deeper investigation into the development of structural composite materials that can achieve the “thin, broad, light, and strong” characteristics for electromagnetic wave absorption. The selection of high-performance polymer matrices significantly influences the mechanical properties and wave-transmitting capabilities of the composite materials. While the choice of a single absorbent and the blending methods of multiple absorbents primarily affect the absorption performance of the composite, relying solely on the type of absorbent is no longer sufficient to meet the requirements. Therefore, structural design is employed to enhance the absorption performance of composite materials while also improving their mechanical load-bearing capacity. However, several issues persist: (1) Current research on absorbent composite materials with resin matrices focuses on enhancing absorption performance but must also consider other factors such as mechanical load-bearing performance, weight, thickness, and cost. (2) The selection of absorbents is crucial for the absorption performance of composite materials, yet the blending methods and achieving the ideal dispersion of absorbents within the resin matrix are key areas for future research. (3) Structural design has led to the development of multi-layer structures, multi-layer sandwich structures (with foam cores, honeycomb cores, corrugated cores, etc.), and FSS. These designs can significantly increase the absorption performance of structure–functional integrated materials, but further optimization can be pursued by combining different types of sandwich structures to achieve better absorption effects. (4) The design of frequency-selective surfaces can markedly improve the absorption performance of composite materials, but it does not enhance the mechanical properties of the materials—a problem that requires continued research. The integration of structure and absorption functionality in composite materials can improve absorption performance while also enhancing the mechanical properties of the composite.

The investigation of diverse structural absorbent composite materials has facilitated an analysis and discussion of the absorption characteristics of these structures as applied to designed composites. The future development of these composites will balance considerations of absorption efficacy with economic and weight constraints. The design of FSS structures has demonstrated a significant enhancement in absorption performance; however, their mechanical property limitations warrant further investigation. Moreover, composites formed by combining different structural types (honeycomb with foam sandwich, FSS with foam sandwich, etc.) can show better absorption capacity, offering a promising avenue for the innovation of new configurations in the future.

## Data Availability

Data availability is not applicable to this article as no new data were created or analyzed in this study.
